# On the Use of Belladonna in Cholera

**Published:** 1866-07

**Authors:** J. A. Young

**Affiliations:** Monmouth, Ill.


### ARTICLE XXIII.

EDITOR EXAMINER:—The following letter, from our intelligent and ingenious friend, Dr. JOHN A. YOUNG, of Monmouth, so clearly expresses his views, as to the theory and therapeutics of cholera, that I am induced to think that it would be interesting to your readers. Should you agree with me in this respect, I hope to see it in your valuable periodical.

Respectfully,

W. H. BYFORD.

MONMOUTH, May 17th, 1866.

PROF. W. H. BYFORD,

*Dear Friend:*—Will you be kind enough to bear with me whilst I may inflict upon you a long, and perhaps profitless, communication. My object is to present a few thoughts and suggestions that have occurred to me, upon that *much-abused* subject—the treatment of cholera. And, to come directly and at once to the point, to direct your attention to, and ask your opinion in regard to, the use of Belladonna, or its active principle, Atropine, in impending collapse, or, after this stage has set in—administering by hypodermic injection. I do not know whether this has ever been proposed or acted on by any one or not; if it has, I am not aware of the fact.

I will endeavor, in as brief and concise manner as possible, to state to you the process of thought that led me to the conclusion that atropine might, at least, prove an useful auxiliary in that condition. I will admit that I was, perhaps, *too* favorably impressed with the theory of Dr. CHAPMAN, of London, when it was first announced, owing, doubtless, to the fact that I was at that time much interested in studying the works of BROWN-SEQUARD, upon the pathology of the nervous centres. The subsequent reports made by Dr. C. and other physicians of Southampton of the trial of his method rather confirmed me, although the cases were too few in number to establish it.

Since that report, I have carefully read the lectures of Dr. GEO. JOHNSON, Prof. of Med. in Kings College. In speaking of Dr. CHAPMAN'S theory, he designates it as "a speculative web, spun from the projector's brain." What special part of the theory he views in this light, he does not state; but, in advocacy of his *own* theory of the cause, pathology, and treatment of cholera—which he puts forth with great boldness—asserts, as an incontrovertible fact, that collapse is *not* occasioned by depletion from loss of serum, but by *spasmodic contraction* of the capillary branches of the pulmonary artery, preventing due oxygenation of the blood, etc., etc.

He contends that a morbid material is conveyed by the *blood*, causing irritation and consequent spasm, therefore we must *eliminate.*

But, if his theory of collapse is correct—and which he thinks and *probably proves* to be correct—then why not resort to some means calculated to act directly on the vaso-motor system.

If we can only protect the nervous system, or so modify its action as to render it insensible to the influence of the morbid material, prevent contraction, and maintain the circulation, surely we have attained an important point.

Practically, it is of but little importance to us to know whether the essential cause of the disease has acted primarily upon the nervous centres, or has been conveyed to them by the blood. The all-important question is, what is the true condition present in a given case, and how shall we remedy it, leaving the abstract question of the essential cause to be settled at leisure.

One authority more:—In the last No. of the *London Lancet*, we have a lecture from Dr. MACLEAN, Deputy-Inspector-General, on the treatment of cholera; and in referring to the theory of Dr. CHAPMAN, speaks of it as an "ingenious one;" and although he differed materially from Dr. C., as to the mode in which the disease is propagated, says he would "gladly give it a fair trial, and, had the disease appeared in Victoria Hospital, was prepared to do so." The mode of propagation to which he refers, I understand to be Dr. C.'s position, that the hyperæmic condition of the nervous centres presiding over the bowels is caused by "*excessive external heat.*" This he denies, and adduces the proof.

To my mind, however, this objection does not materially affect the question.

*Does this hyperæmic condition actually exist, let the cause be what it may?*

If so, then, in accordance with the theory of BROWN-SEQUARD, we have, in belladonna, a remedy of great potence in such condition.

I would also adduce, in favor of the idea, the well-known facts of the effect of full doses of this drug upon the capillaries of the skin, producing engorgement, with redness and heat, simulating scarlatina, and just the opposite of collapse.

Yours, &c.,

J. A. YOUNG.

